# Virtual bronchoscopic navigation with intraoperative cone-beam CT for the diagnosis of peripheral pulmonary nodules

**DOI:** 10.1186/s12890-024-02930-0

**Published:** 2024-03-20

**Authors:** Jisong Zhang, Enguo Chen, Shan Xu, Li Xu, Huihui Hu, Liangliang Dong, Kejing Ying

**Affiliations:** Department of Pulmonary and Critical Care Medicine, Regional Medical Center for National Institute of Respiratory Disease, Sir Run Run Shaw hospital of Zhejiang University, 310016 Hangzhou, China

**Keywords:** Cone beam CT, Peripheral pulmonary nodules, Transbronchial biopsy, Virtual bronchoschopic navigation

## Abstract

**Objective:**

Transbronchial biopsy is a safe manner with fewer complications than percutaneous transthoracic needle biopsy; however, the current diagnostic yield is still necessitating further improvement. We aimed to evaluate the diagnostic yield of using virtual bronchoscopic navigation (VBN) and cone-beam CT (CBCT) for transbronchial biopsy and to investigate the factors that affected the diagnostic sensitivity.

**Methods:**

We retrospectively investigated 255 patients who underwent VBN-CBCT-guided transbronchial biopsy at our two centers from May 2021 to April 2022. A total of 228 patients with final diagnoses were studied. Patient characteristics including lesion size, lesion location, presence of bronchus sign, lesion type and imaging tool used were collected and analyzed. Diagnostic yield was reported overall and in groups using different imaging tools.

**Results:**

The median size of lesion was 21 mm (range of 15.5–29 mm) with 46.1% less than 2 cm in diameter. Bronchus sign was present in 87.7% of the patients. The overall diagnostic yield was 82.1%, and sensitivity for malignancy was 66.3%. Patients with lesion > 2 cm or with bronchus sign were shown to have a significantly higher diagnostic yield. Four patients had bleeding and no pneumothorax occurred.

**Conclusion:**

Guided bronchoscopy with VBN and CBCT was an effective diagnostic method and was associated with a high diagnostic yield in a safe manner. In addition, the multivariant analysis suggested that lesion size and presence of bronchus sign could be a predictive factor for successful bronchoscopic diagnosis.

## Summary at a glance

Our study showed that the combined use of VBN and CBCT enabled the bronchoscopic diagnosis of PPLs and was associated with an overall high diagnostic yield of 82.4%. In addition, the multivariate analysis suggested that lesion size and presence of bronchus sign was a significant predictive factor for successful bronchoscopic diagnosis.

## Introduction

As lung cancer is the primary cause of malignancy-related mortality, early detection and evaluation are essential to considerably raise the 5-year survival rate compared to a late-stage diagnosis [1]. With the development and widespread use of computed tomography (CT) screening, a greater number of peripheral pulmonary nodules have been identified, necessitating further diagnostic evaluation [2]. However, it remains challenging in navigating and sampling of PPLs due to poor access to target lesions [3]. Conventional bronchoscopy has a limited diagnostic yield for PPLs, which has been reported a rather low sensitivity from 14 to 63% and even lower for those less than 20 mm in diameter [4, 5].

To address this issue, a variety of techniques such as ultrathin bronchoscopy, radial probe endobronchial ultrasonography (r-EBUS), electromagnetic navigation bronchoscopy (ENB), virtual bronchoscopic navigation (VBN), and combinations of these techniques are available [6, 7]. ENB and VBN, as one of the dominant techniques of navigation bronchoscopy, have been widely used clinically in the diagnosis of PPLs in recent years. When compared to percutaneous transthoracic needle biopsy (PTNB), navigation bronchoscopy has the advantages of being suitable for various types of peripheral lesions and having a lower complication rate; however, it has a comparable lower diagnostic yield [8–12]. The diagnostic yield guided by ultrathin bronchoscopy and VBN is highly variable (65-81%) [13–15], with an overall diagnostic yield of ∼ 70%. Whereas CT-guided PTNB has an overall yield of more than 90% for diagnosing lung lesions [16, 17]. In this aspect, there is still a need and potential to empower physicians in accessing the peripheral lung nodules and improving the diagnostic accuracy of navigational bronchoscopy.

Cone-beam CT (CBCT), one of the more recent innovations, is being used to navigate to PPLs. During the bronchoscopy procedure, CBCT is a technique that offers 3D images of the lesion and/or tool location for additional guidance and real-time confirmation [6]. According to a prospective pilot trial, adding CBCT to ultrathin bronchoscopy and r-EBUS could boost the diagnostic yield for PPLs from 50–75%^18^. A prospective study of 29 patients by Ali et al. [19] demonstrated a diagnostic yield of 90% for PPLs using the combination of an ultrathin bronchoscope, VBN and CBCT. However, the effectiveness of CBCT-guided VBN approach has only been evaluated in small sample size (< 30 patients) and further investigation is needed.

In this larger-scale study, we retrospectively evaluated the diagnostic yield of the diagnostic method of VBN and CBCT guided transbronchial biopsy for PPLs. In addition, we investigated and analyzed the factors that affected the diagnostic sensitivity of VBN-guided biopsy, such as lesion size, location, and combined use of other assisted techniques like r-EBUS and ROSE.

## Methods

### Patients

**Fig. 1:**
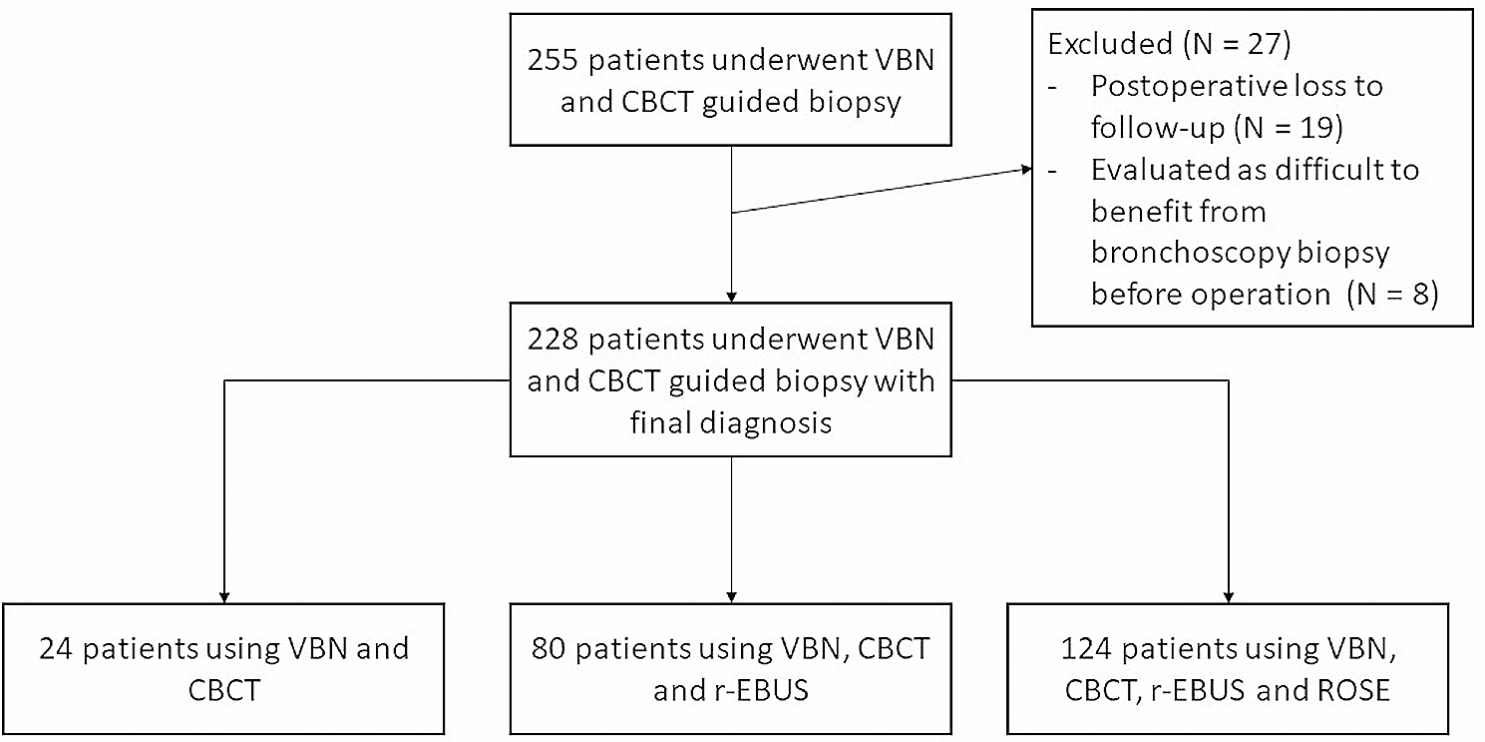
Flow chart

Between May 2021 and April 2022, the medical records of 255 patients (147 males and 108 females) who underwent VBN and CBCT guided transbronchial biopsy at Respiratory Endoscopy Center of Sir Run Run Shaw Hospital (including two medical centers at Qingchun and Qiantang branches) were retrospectively reviewed. Patients inclusion criteria were as follows: (a) they had one or more peripheral pulmonary lesions suggestive of lung cancer, (b) lesions between 10 and 30 mm in diameter(The diameter mentioned in the article is the average of the maximum and minimum diameters), (c) the lesions was pure ground-glass, part-solid or solid nodules, (d) the lesion was surrounded by lung parenchyma and was not visible in the bronchial lumen above the segment. Patients exclusion criteria were as follows: (a) patients were lost to follow-up after biopsy, (b) it was judged before the operation that the patients were difficult to benefit from bronchoscopy biopsy (such as high risk of bleeding due to perivascular wrapping around the lesion, difficulty in reaching the airway adjacent to the lesion due to previous lung surgery, etc.). Due to postoperative loss to follow-up (*N* = 19) and difficult to benefit from bronchoscopy biopsy (*N* = 8), 27 patients in total were excluded. 228 patients were finally investigated in this study. Operations were performed by different pulmonologists. The use of all tools and the amount of sampling were decided by the endoscopy team after considering factors such as safety and lesion characteristics. The flow chart of the study was shown in Fig. 1. The study received approval from the institutional review board of Sir Run Run Hospital. The authors confirm that their study was performed in accordance with the Declaration of Helsinki. Patient consent was waived by the Institutional Review Board due to retrospective nature of the study.

### Virtual bronchoscopy navigation

All patients underwent peripheral pulmonary nodules biopsy under the guidance of a VBN system (Lungpro; Broncus Medical Inc.). Before treatment, each patient underwent a thin-section chest CT scan from the apex of the lung to the diaphragm during inspiration breath-hold. CT scans were reconstructed in 1-2.5 mm thick slices at 1 mm intervals. The virtual bronchoscopy image was automatically created using CT imaging once the pulmonologist had set the target lesion. A predetermined path from the tracheal bifurcation to the leading bronchi of the target lesion could be employed for navigation. When it comes to poor reconstruction of bronchus, pulmonologists may need to manually trace the path of supplementary airways.

### CBCT acquisition

A ceiling-mounted angiography system (Artis zee.ceiling; Siemens Healthineers, Forchheim, Germany) was used throughout the bronchoscopy. To confirm the “tool-in-lesion”, CBCT imaging was obtained during inspiration breath-hold using a 6-second acquisition protocol with 400 projection images acquired over a 200-degree rotation. After acquisition, the reconstruction would be completed automatically on a dedicated workstation (*syngo* X Workplace, Siemens Healthineers) with cross-sectional three-dimensional images displayed. Additional CBCT scans were carried out when the tool position required to be adjusted.

**Fig. 2:**
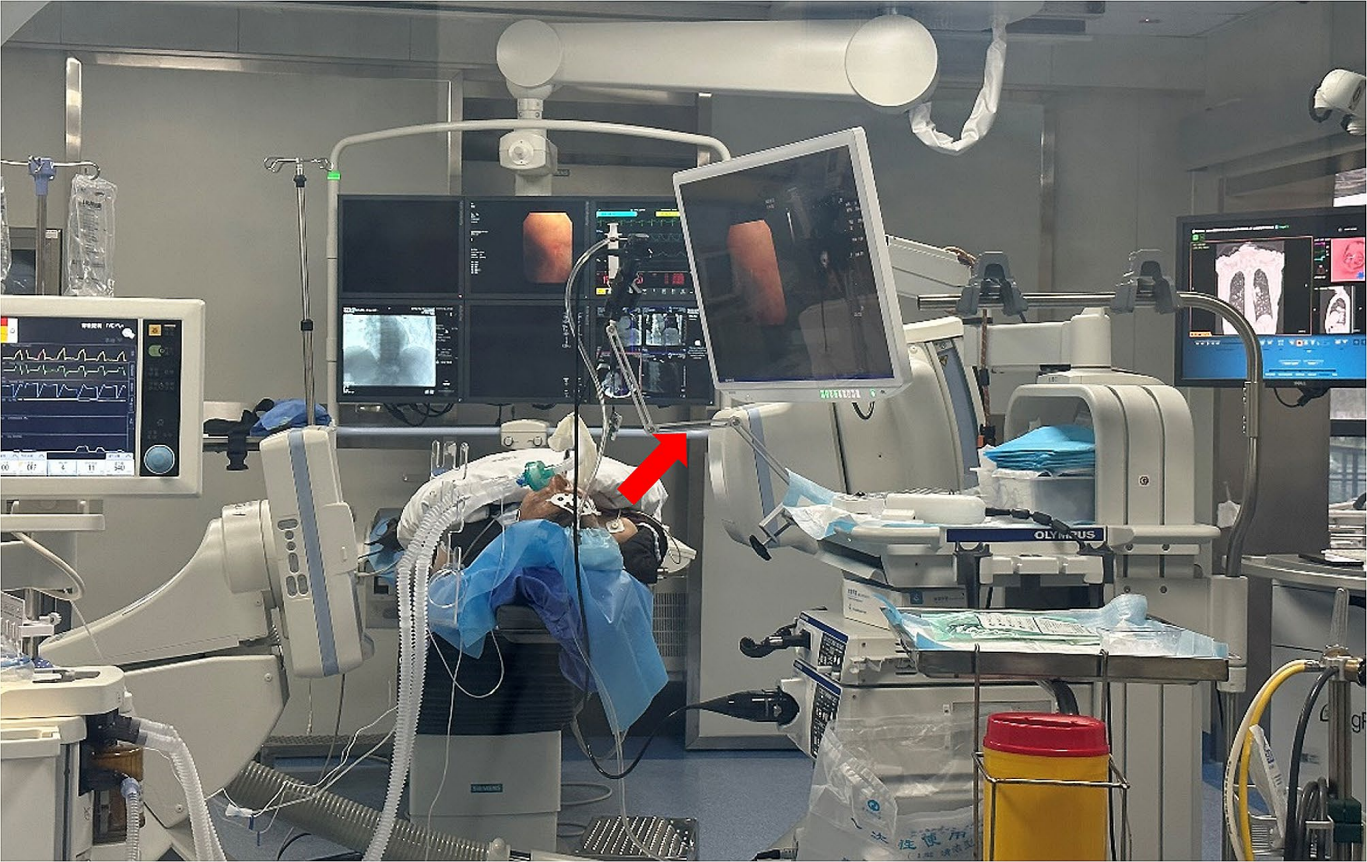
Room set up with fixed c-arm. The red arrow corresponds to the arm that holds the bronchoscopy when performing the CBCT scan

During CBCT scan, the bronchoscope was fixed to an arm that was attached to the bronchoscopy room, so that all personnel could leave the room (Fig. 2). A pulmonologist was only necessary to hold the bronchoscope only when the fixation was weak due to a sharp angle of a rarely accessible lesion.

### Procedures

**Fig. 3:**
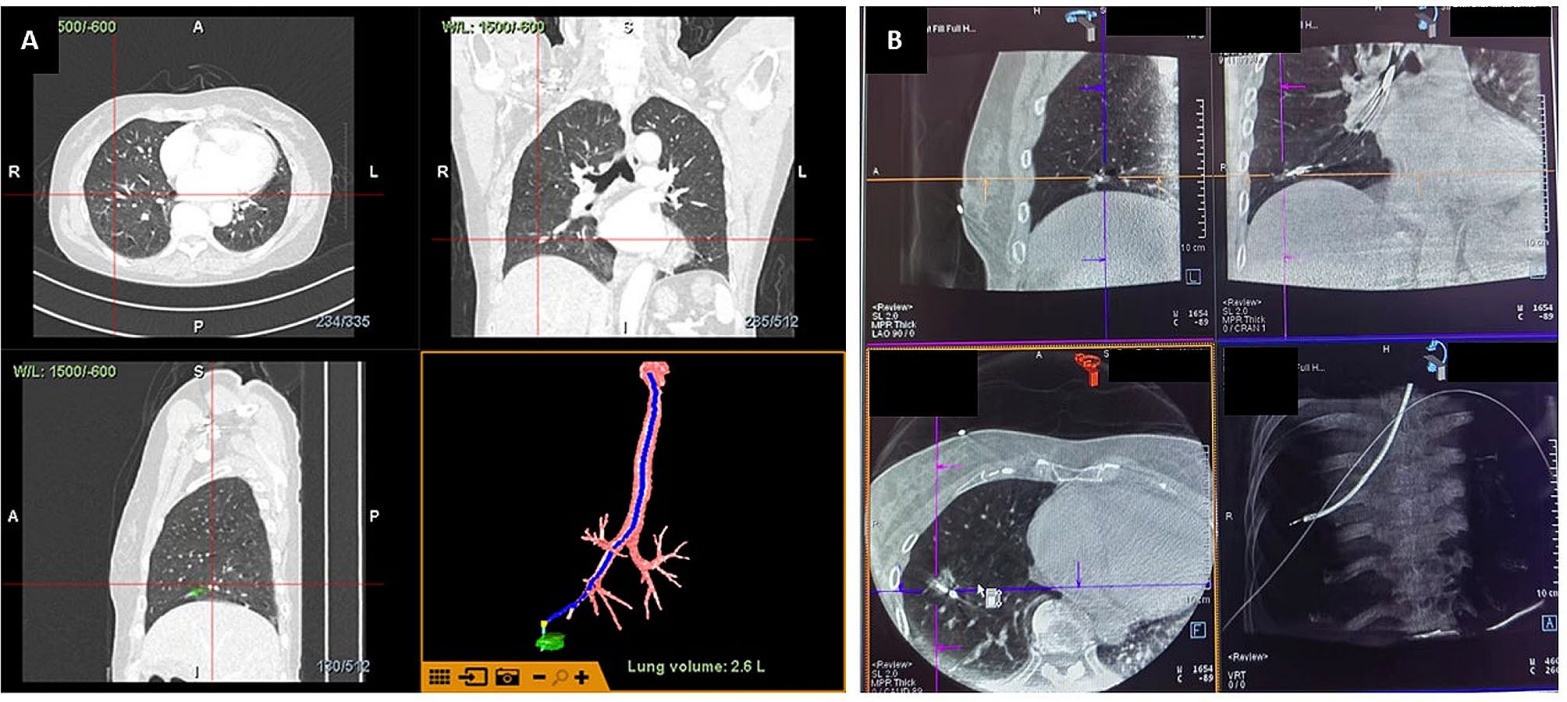
(**A**) VBN showing the planned path reaching the target lesion; (**B**) CBCT images from 3 dimensions (vertical, frontal, and sagittal) view showing tip of device within the target. VBN: virtual bronchoscopy navigation; CBCT: cone beam computed tomography

All patients underwent general anesthesia, endotracheal intubation, and continuous intraoperative maintenance with dextromethorphan, remifentanil, and propofol were administered to all patients. A flexible bronchoscope with a working channel diameter of less than 2.0 mm (Bf-P290; Olympus; external diameter, 4.1 mm) was introduced along the airway to briefly observe the tracheobronchial lumen. To keep the airway clean and the endoscopic view clear, if there are abnormal new organisms or sputum accumulation, remove the airway secretions. By comparing the direct vision and navigation images provided by the VBN system, the bronchoscope was advanced as far as possible into the target bronchi (Fig. 3-A). Before a CBCT confirmation, r-EBUS (UM-S20-17 S; Olympus) was also often additionally used to confirm that the probe had reached the lesion. Whether to use it depends on the evaluation of the lesion by the bronchoscopist, including the size, location, and presence or absence of bronchus signs. Of course, it is also related to the habit of bronchoscopist. Guide sheath is not used during r-EBUS confirmation. After the biopsy tool or r-EBUS was in the correct position of the target lesion, a CBCT scan was performed to confirm the relationship of the tool to the lesion, where 3D confirmation of lesion access was available (Fig. 3-B). Based on the results of the CBCT, the pulmonologist decided whether to proceed directly to the biopsy specimen collection or to readjust the biopsy tool to a superior position. Before to confirm that the probe had reached the lesion. Intermittent rapid on-site evaluation (ROSE) results of samples were used to determine upon the need for additional sampling.

### Diagnosis

**Fig. 4:**
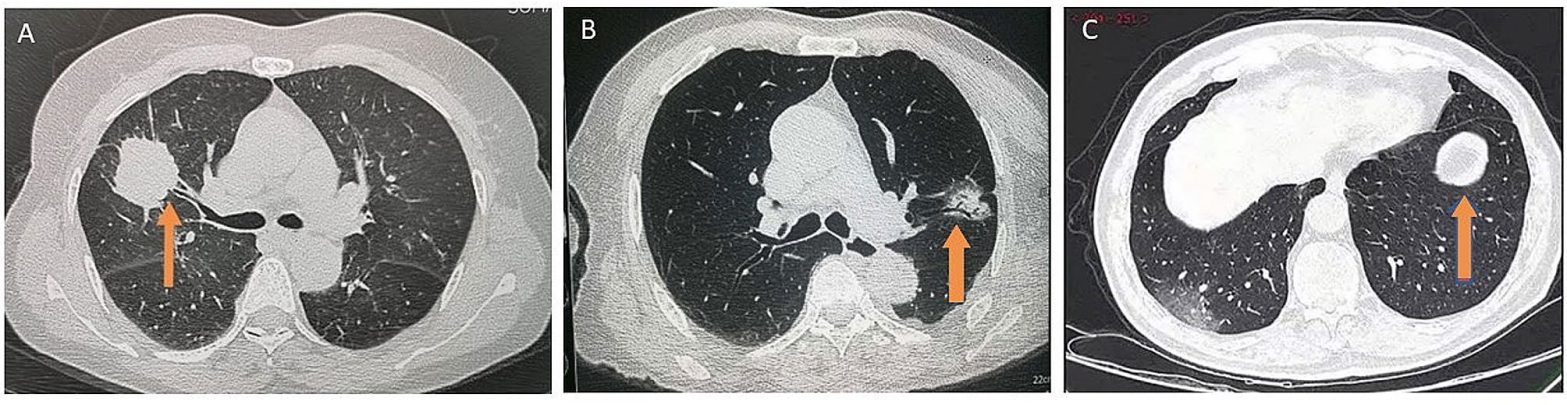
CT imaging shows (**A**) the presence of bronchus truncation sign (**B**) the presence of bronchus passage sign (**C**) no bronchus sign

In this study, the presence of bronchus sign was defined into bronchus truncation sign (Fig. 4-A), bronchus passage sign (Fig. 4-B) and no bronchus sign (Fig. 4-C). Bronchus truncation sign was defined when CT image shows that the bronchial ostia is truncated by the distal lesion, frequently indicating tumor tissue penetration into the airway. We defined bronchus passage sign as CT image that shows the bronchial passage across the lesion, indicating that the tumor tissue has not invaded the airway or simply the airway mucosa. According to the experience in practice, the diagnostic rate of upper top area and other areas were counted separately. The upper top area was defined as LB1 + 2 and RB1. As an important evaluative parameter, diagnostic yield was defined as the proportion of patients from whom diagnostic samples were obtained. If the pathologic report returns a neoplastic lesion (either a benign tumor or a malignant tumor), defined as a positive diagnosis. We are more cautious with those cases whose final pathologic report is infiltration of inflammatory cells in lung. For those who return with inflammation pathology, we will continue to follow up for no less than three months to exclude false positive results based on subsequent changes in lesion.

### Statistical analysis

All data were analyzed using SPSS 22.0 statistical software. Categorical data were summarized as frequencies and percentiles using the chi-square test or Fisher’s exact probability method. For continuous variables, data are presented as the mean ± standard deviation for variables normally distributed and as median with interquartile range (IQR) for skewed variables. Normally distributed continuous variables were compared using a 2-sided Student’s *t* test, and the Mann-Whitney U test was used for nonparametric variables. Statistical significance was set to 5%.

## Results

A total of 228 patients (133 males and 95 females) with median age of 64 years (range of 56–72 years) with final diagnosis were included in this study. All the patients received navigation of VBN and CBCT, among which 89.5% received r-EBUS for lesion confirmation at the same time and 54.4% had immediate ROSE evaluation. The average diameter of peripheral pulmonary nodules was 21 mm (range of 15.5–29 mm), and 46.1% of them were less than 2 cm in diameter. There were 12.3% of the patients with no bronchus sign, 30.7% with bronchus passage sign and 57.0% with bronchus truncation sign. In terms of lesion type, 61.4% was solid, 31.6% was part-solid and 7% was pure GGO. Patient baseline characteristics were described in Table 1.

**Table 1:** Baseline characteristics

Characteristics (*N* = 228)	Values
Age, years	
Median (range), mm	64 (56–72)
Age > 70, N (%)	157 (68.9%)
Gender, N (%)	
Male	133 (58.3%)
Female	95 (41.7%)
Lesion size, median (range)	
Average diameter, mm	21 (15.5–29)
Diameter ≤ 2 cm, N (%)	105 (46.1%)
Lesion location, N (%)	
Left: 94; Right: 134	
Upper	112
Middle	20
Lower	96
Bronchus sign, N (%)	
Yes	200 (87.7%)
Bronchus passage sign	130 (57.0%)
Bronchus truncation sign	70 (30.7%)
No	28 (12.3%)
Lesion type, N (%)	
Solid	140 (61.4%)
Part-solid	72 (31.6%)
Pure GGO	16 (7.0%)
Imaging tool, N (%)	
VBN + CBCT	24 (10.5%)
VBN + CBCT + r-EBUS	80 (35.1%)
VBN + CBCT + r-EBUS + ROSE	124 (54.4%)

Values are median (range), or N (%)

Abbreviations: GGO: ground-glass opacity; CBCT: cone-beam CT; r-EBUS: radial probe endobronchial ultrasonography; ROSE: rapid on-site evaluation

The overall diagnostic yield was 82.1% (see diagnostic yield of bronchoscopy by categorical parameters Table 2). The diagnostic yield was categorized by different parameters and analyzed by groups. The results showed that patients with lesions > 2 cm or with bronchus sign were associated with a significantly higher diagnostic yield. Patients with lesion located in the upper top area were shown to have a significantly lower diagnostic yield. A total of 4 patients (1.75%) had bleeding after biopsy who required post pituitary pigment to stop bleeding, and no pneumothorax occurred.

**Table 2:** Diagnostic yield of bronchoscopy by categorical parameters

Characteristics (*N* = 228)	Diagnostic yield	*P* value
Lesion size		0.030
≤ 2 cm	75.24% (79/105)	
> 2 cm	90.24% (111/123)	
Lesion location		0.002
Upper top (LB1 + 2/RB1)	70.27% (26/37)	
Others	85.86% (164/191)	
Bronchus sign		0.001
Yes	90.5% (181/200)	
Bronchus passage sign	88.46% (115/130)	
Bronchus truncation sign	94.29% (66/70)	
No	32.14% (9/28)	
Lesion type		0.523
Solid	87.14% (122/140)	
Subsolid	77.27% (68/88)	
Part-solid	83.33% (60/72)	
Pure GGO	50% (8/16)	
Imaging tool		0.093
VBN + CBCT	83.33% (20/24)	
VBN + CBCT + r-EBUS	76.25% (61/80)	
VBN + CBCT + r-EBUS + ROSE	87.90% (109/124)	
Overall	82.1%	

Abbreviations: LB: left lobe; RB: right lobe; GGO: ground glass opacity; CBCT: cone-beam CT; r-EBUS: radial probe endobronchial ultrasonography; ROSE: rapid on-site evaluation

Through transbronchial lung biopsy, 187 cases were diagnosed, of which 124 were neoplastic lesions and 63 were non-neoplastic lesions. For non-neoplastic lesions, the patient would require at least 3-month follow-up to exclude the possibility of false positive. The sensitivity of malignancy was 66.3%. Specific diagnoses are described in Table 3. As a subgroup analysis, use of VBN + CBCT had a diagnostic yield of 83.33% and in the group using VBN + CBCT + r-EBUS, the diagnostic yield was 76.25%. With the additional use of ROSE, the diagnostic yield reached 87.90% (see Table 4). However, there was no statistical difference between these three groups.

**Table 3:** Final diagnoses

Results	Numbers
Pathological findings	187
Neoplastic	124
Lung adenocarcinoma	88
Squamous cell carcinoma of the lung	14
Small cell carcinoma	6
Metastatic carcinoma	3
Large cell neuroendocrine carcinoma	2
Undifferentiated carcinoma	2
MALT lymphoma	2
Sclerosing pulmonary cell tumor	1
Adenosquamous cell carcinoma	1
Synovial sarcoma	1
Leiomyosarcoma	1
Carcinoid	1
Adrenal origin tumor	1
Salivary gland origin tumor	1
Non-neoplastic	63
Inflammation	31
Tuberculosis	13
Cryptococcal pneumonneumococcosis	6
Organizing pneumonia	4
Lung abscess	3
Pulmonary aspergillosis	3
Pulmonary sarcoidosis	2
Alveolar proteinosis	1

**Table 4:** Subgroup analysis of diagnostic yield in different imaging tools

Characteristics (*N* = 228)	VBN + CBCT (*N* = 24)	VBN + CBCT + r-EBUS (*N* = 80)	VBN + CBCT + r-EBUS + ROSE (*N* = 124)	F/X^2^	*P* value
Diagnostic yield	83.33% (20/24)	76.25% (61/80)	87.90% (109/124)	4.755	0.093
Lesion size					
≤2 cm	86.67% (13/15)	63.33% (19/30)	78.33% (47/60)	3.642	0.162
>2 cm	77.78% (7/9)	84% (42/50)	96.88% (62/64)	6.999	0.030
Lesion type					
Pure GGO	-	50% (4/8)	57.14% (4/7)	0.077	0.782
Part-solid	90% (9/10)	80% (16/20)	83.33% (35/42)	0.480	0.787
Solid	84.62% (11/13)	78.85% (41/52)	93.33% (70/75)	5.834	0.054
Bronchus sign					
No	42.86% (3/7)	16.67% (2/12)	44.44% (4/9)	2.311	0.315
Bronchus truncation sign	100% (12/12)	93.94% (31/33)	92% (23/25)	0.977	0.614
Bronchus passage sign	100% (5/5)	80% (28/35)	91.11% (82/90)	3.726	0.155

Abbreviations: GGO: ground-glass opacity; CBCT: cone-beam CT; r-EBUS: radial probe endobronchial ultrasonography; ROSE: rapid on-site evaluation

## Discussion

The present study showed that the combined use of VBN and CBCT enabled the bronchoscopic diagnosis of PPLs and was associated with a high diagnostic yield. The overall diagnostic yield was 82.4%. In addition, lesion size and presence of bronchus sign was a significant predictive factor for successful bronchoscopic diagnosis. To the best of our knowledge, this is the largest report on the specific combination of VBN, CBCT, r-EBUS and ROSE for diagnostic yield of bronchoscopy for PPLs.

Navigational bronchoscopy platforms, endobronchial ultrasound, CBCT and especially their combined use was rapidly evolving and showed a potential of increasing the reach for peripheral lung lesions. Previous studies reported a diagnostic yield of 67.1 − 74% for VBN-guided bronchoscopy [20, 21]. Compared with VBN alone, Asano et al. [22] suggested that use of X-ray fluoroscopy on top of conventional VBN guided biopsy with r-EBUS confirmation increases the diagnostic yield from 76.9 to 85.9%. As an alternative add-on tool, CBCT has been demonstrated for its valuable benefits on tool-in-lesion confirmation and position readjustment, especially for the lesions that are not visible under fluoroscopy [23]. In a study of eighty-nine patients, the addition of CBCT imaging to the ENB method results in a 35.5% absolute increase in navigation success and an overall diagnostic accuracy of 72.4% 24. In combined use of CBCT and ENB, Pritchett et al. [25] also suggested a diagnostic yield of 83.7% using the intraprocedural image fusion with augmented fluoroscopy. Ali et al. [19] further reported a very high yield in the diagnosis of PPLs guided by CBCT and VBN, which is 90%; however, the sample size is limited to 29 patients. In our study, we retrospectively investigated 228 patients and found a comparable diagnostic yield of 87.9% when using VBN, r-EBUS, CBCT for guidance and confirmation, and ROSE for intra-procedural evaluation. For patients with lesion larger than 2 cm or solid lesion or with the presence of bronchus sign, the diagnostic yield could reach higher than 90%. In this way, the overall diagnostic yield of CT-guided PTNB, which is greater than 90% for the diagnosis of lung lesions [16, 17], might be approached or even achieved by the VBN-CBCT-guided bronchoscopic method.

Among the existing navigational bronchoscopy systems, CBCT navigation is the only real-time navigation technology based on intraoperative 3D images. Although r-EBUS is similarly real-time, the lack of resolution of GGOs, the single imaging dimension, and numerous confounding variables (such as hemorrhage, local irrigation with normal saline, etc.) limit its clinical application. As a significant on-site auxiliary confirmation technique, ROSE has been shown to enhance the diagnostic rate; nonetheless, there is a lack of acknowledged consensus criteria for the ROSE assessment, and various evaluators may interpret the same cytology smear differently. The use of ROSE as an additional on-site confirmation tool is also constrained by its inability to control the depth and orientation of the intraoperative operation in real time. The current ENB or VBN technology is primarily guided by preoperative CT images. Under intraoperative anesthesia, changes in lung volume, atelectasis, and pulmonary nodule displacement brought on by respiratory exercise can result in changes in the spatial position relationship of the target lesion, which can affect the diagnostic rate. Previous studies have supported the hypothesis that preoperative CT and intraoperative CT display different nodule positions in actual practice [26]. From this perspective, CBCT can monitor the diagnosis and treatment process in real time and determine the interaction between the biopsy tool and the target lesion, so as to increase the accuracy of the diagnosis. In addition, studies [27, 28] showed that GGOs are perfectly visible and able to localize on intraoperative CBCT images. Chen et al. [29] further reported that CBCT imaging can provide real-time confirmation of “tool-in-lesion” during transbronchial ablation so that the device position can be adjusted accordingly based on 3D images of tool position with respect to GGO nodules.

There are limitations to this study. First, this is a retrospective study that allows for indications of bias in subgroup analysis. In result, the diagnostic yield of VBN and cone-beam CT alone was better than that of the combined r-EBUS group. The reasons need to be considered for selection bias. For some lesions that are more easily reached, we may prefer to perform biopsy directly rather than r-EBUS for further confirmation. In addition, due to poor vision, sputum accumulation, or the need for BALF prior to biopsy, it may also be necessary to flush the bronchial lumen with saline before arrival. This may interfere with r-EBUS images, especially for ground glass lesion [3, 7]. Thus, a multi-center prospective study is warranted. Second, some pulmonologists were not getting extra CBCT imaging to record the information of “tool-in-lesion”. Therefore, we do not have the measurement of technical success, which we do believe is related to the clinical value of real-time CBCT confirmation. Furthermore, the effective dose of radiation employed for every patient was not measured in this study. As with the use of holding arms, pulmonologists are free from radiation exposure. For the radiation exposure to the patient, Casal et al. [18] reported that body radiation doses were between 8.6 and 23 mSv resulting from CBCT, which is associated with an acceptable radiation dose. The evaluation of technical success and radiation exposure will be investigated in our future work.

In conclusion, guided bronchoscopy with VBN and CBCT was an effective diagnostic method and was associated with a high diagnostic yield. In addition, the results of subgroup analysis also suggested that lesion size, presence of bronchus sign and lesion location could be a predictive factor for successful bronchoscopic diagnosis. However, further prospective randomized studies are necessary to clarify the benefit of this diagnostic method.

## Data Availability

The datasets used and/or analysed during the current study are available from. the corresponding author on reasonable request.

## Abbreviations

CBCT: Cone-Beam Computed Tomography
CT: Computed Tomography
ENB: Electromagnetic Navigation Bronchoscopy
GGO: Ground-Glass opacity
IQR: Interquartile Range
PTNB: Percutaneous Transthoracic Needle Biopsy
r-EBUS: Radial Probe Endobronchial Ultrasonography
ROSE: Intermittent Rapid On-Site Evaluation
VBN: Virtual Bronchoscopic Navigation
